# Evaluation of the Isoflavone Genistein as Reversible Human Monoamine Oxidase-A and -B Inhibitor

**DOI:** 10.1155/2016/1423052

**Published:** 2016-03-28

**Authors:** Najla O. Zarmouh, Samia S. Messeha, Faisel M. Elshami, Karam F. A. Soliman

**Affiliations:** College of Pharmacy & Pharmaceutical Sciences, Florida A & M University, Tallahassee, FL 32307, USA

## Abstract

Monoamine oxidases inhibitors (MAOIs) are effective therapeutic drugs for managing Parkinson's disease (PD) and depression. However, their irreversibility may lead to rare but serious side effects. As finding safer and reversible MAOIs is our target, we characterized the* recombinant human* (*h*) MAO-A and MAO-B inhibition potentials of two common natural isoflavones, genistein (GST) and daidzein (DZ) using luminescence assay. The results obtained showed that DZ exhibits partial to no inhibition of the isozymes examined while GST inhibited* h*MAO-B (IC_50_ of 6.81 *μ*M), and its* h*MAO-A inhibition was more potent than the standard deprenyl. Furthermore, the reversibility, mode of inhibition kinetics, and tyramine oxidation of GST were examined. GST was a time-independent reversible and competitive* h*MAO-A and* h*MAO-B inhibitor with a lower *K*_*i*_ of* h*MAO-B (1.45 *μ*M) than* h*MAO-A (4.31 *μ*M). GST also inhibited* h*MAO-B tyramine oxidation and hydrogen peroxide production more than* h*MAO-A. Docking studies conducted indicated that the GST reversibility and* h*MAO-B selectivity of inhibition may relate to C5-OH effects on its orientation and its interactions with the threonine 201 residue of the active site. It was concluded from this study that the natural product GST has competitive and reversible MAOs inhibitions and may be recommended for further investigations as a useful therapeutic agent for Parkinson's disease.

## 1. Introduction

Neurological disorders such as Parkinson's disease (PD) and major depressive disorder (MDD) are associated with brain neurotransmitters depletion, particularly dopamine (DA), serotonin (5-HT), and norepinephrine (NE). MDD comorbidities may affect 72% of PD patients [1]. However, the currently available drugs for PD patients have wearing-off complications and for MDD are unsuccessful in at least 40% of the patients [2, 3]. In depressive and aging PD patients, the activity of brain monoamine oxidase (MAO) is significantly elevated compared to healthy individuals [4, 5]. Moreover, in the aging population, improving brain neurotransmitters and reducing MAO activities by using MAO inhibitors (MAOIs) were proven beneficial whereas other antidepressants mechanisms appear less effective [6].

MAO exists in two isoforms, MAO-A, and MAO-B [7]. The therapeutic value of the reversible inhibitors of MAO-A (RIMA) for depression has been firmly established [8] while MAO-B inhibitors (MAO-BIs) are used in PD. MAO-BIs maintain a higher level of DA from its precursor L-dopa in PD patients and may even benefit Alzheimer's disease (AD) [9, 10]. Deprenyl (DEP), a current MAO-BI used for PD, was recently approved as transdermal patches (Emsam®) for MDD treatment to become the most prevalent MAOI useful for the elderly [6].

In the brain, the mitochondrial bound flavin-containing amine oxidoreductase (MAO; EC 1.4.3.4) works to catalyze monoamine neurotransmitters oxidative deamination to maintain homeostasis. MAO-A catalyzes NE, DA, and 5-HT, while MAO-B catalyzes DA more specifically [11]. The abnormal elevation of MAO activity is associated with oxidative stress due to their production of hydrogen peroxide (H_2_O_2_) and aldehydes [12, 13]. Glutathione peroxidase and other enzymes become limited in buffering H_2_O_2_, and oxidative stress occurs and rises with the presence of iron, aggravating DNA and lipid membrane damage and contributing to glial and neuronal cell death. Hence, MAO inhibition provides a valuable strategy for the prevention of oxidative stress in addition to its ability in maintaining functional neurotransmitters levels.

The earlier discovered nonselective MAOIs, such as phenelzine, or the current selective MAO-BIs such as DEP have an irreversible inhibitory mechanism [14]. MAO irreversible inhibition may lead to the rare incidences of “the cheese effect” or drug interactions side effects [15]. Recent research had recognized that reversible or competitive inhibition of MAOs might be more important than isoform selectivity or high potency [16]. Therefore, reversible MAO inhibition is possibly the best strategy to prevent the cheese effect reaction caused by tyramine rich food ingestion. Therefore, the higher levels of the substrates or the withdrawal of the reversible inhibitors would allow the inhibitor substitution and, consequently, a faster recovery of the enzyme leading to fewer side effects.

Flavonoids as chromone-containing polyphenolic structures are well-known natural antioxidant agents that may affect the catecholamine synthesis [17, 18]. Apigenin, luteolin, and quercetin are examples of flavonoids with potent MAO inhibitory activities [19]. The flavonoids subclass isoflavones, in particular, were an interesting investigational class of compounds in the last two decades due to their presence in medicinal and nutritional traditional plants.

Genistein (GST) and daidzein (DZ) are two isoflavone analogs naturally found in traditionally used medicinal plants such as bakuchi, soybeans, and red clover [20–22]. Previously, both analogs showed different activities on distinct MAOs sources; DZ showed no significant rat MAO inhibition [23] while GST showed a weak bovine plasma MAO inhibition [24]. In our search for natural MAOIs, GST and DZ were investigated for* recombinant human* MAO isozymes (*h*MAO-A and* h*MAO-B) inhibition with the objective to understand the mechanism of action and structure-activity relationship. By choosing these two structurally close compounds (natural chemical analogs), any difference in their inhibitory activity can disclose the structure-activity relationship between the extra GST functional group and the MAO-A or MAO-B inhibitions. Comparing another compound (e.g., DZ metabolite s-equol) with GST will not enable us to make a clear conclusion. Ultimately, the current study investigates the possibility of identifying safer and reversible natural MAO inhibitors for the possible therapeutic use in the management of PD.

## 2. Materials and Methods

### 2.1. Materials

MAO-Glow*™* Assay kit was obtained from Promega (Madison, WI, USA). Genistein (GST) (purity > 98%) and tyramine HCl were obtained from Santa Cruz Biotechnology Inc. (Dallas, TX, USA). The isozymes* h*MAO-A and* h*MAO-B derived from recombinant baculovirus and their aliquoted active units (U) were obtained from Sigma-Aldrich. Each* h*MAO-A and* h*MAO-B stock was diluted with 10 mM HEPES in cold Hank's Balanced Salt Solution (HBSS) (pH 7.4) and kept in −80°C until use. Daidzein (DZ) (purity ≥ 98%) and selective standard MAOIs including DEP and clorgyline (CLORG) and other materials were also purchased from Sigma-Aldrich (St. Louis, MO, USA).

### 2.2. *h*MAO-A and* h*MAO-B Assay

GST and DZ* h*MAO-A and* h*MAO-B inhibitory effects were tested by measuring the decrease in Arbitrary Light Units (ALU) using MAO-Glow Assay [25] with slight modifications. The initial velocity linearity of the isozymes was validated before the experiments at RT and in the presence and absence of standard DEP. For the assay, GST and DZ were serially diluted with buffer (pH 7.4) to a maximum final concentration of 250 *μ*M each. Ethanol and DMSO were used (<2%) as solvents. In white opaque 96-well plates, 12.5 *μ*L of each analog was plated with 25 *μ*L of* h*MAO-A or* h*MAO-B for a 0.88 U/mL final concentration (0.04 U per reaction). In other wells, reaction buffer substituted each isozyme (to make the blanks) and each analog (to make negative control). After 40 min incubation, 12.5 *μ*L luciferin derivative substrate (LDS) was added for a final concentration of 40 and 4 *μ*M for* h*MAO-A and* h*MAO-B, respectively. After 2 h incubation, the reactions were stopped by adding 50 *μ*L of Reporter Luciferin Detection Reagent (RLDR). Plates were incubated for 30 min to produce luminescence signal and then samples were read on Synergy HTX Multi-Reader (Bio-Tek, USA).

### 2.3. GST Mode of* h*MAO-A and* h*MAO-B Inhibition

#### 2.3.1. Recovery by Dilution Assay

The recovery of both* h*MAO-A and* h*MAO-B after the inhibition by GST was measured by a previously reported preincubation and dilution method [26] with slight modifications using MAO-Glow Assay at RT [24]. Briefly, 100x isozymes (88 U/mL) or buffer (for blanks) was preincubated separately with GST or buffer (for negative controls) at concentrations of 10x IC_50_ and 100x IC_50_ for 40 min at RT (pH 7.4). For positive control, standards at 10x IC_50_ were simultaneously preincubated (0.03 *μ*M CLORG for* h*MAO-A and 1.01 *μ*M DEP for* h*MAO-B). Tests of GST of 4x IC_50_ with 4x of each isozyme was also simultaneously conducted for extra control. All reactions were subsequently diluted 100-fold when mixed with LDS in a 96-well plate to yield final concentrations of GST of 0.1x IC_50_ and 1x IC_50_. The microplates were then incubated for 30, 40, 50, and 60 min. RLDR was added, and luminescence was measured as mentioned above.

#### 2.3.2. Michaelis-Menten

The modes of MAO inhibitory effects of GST were analyzed by Michaelis-Menten kinetics using MAO-Glow Assay. Standard DEP was used to validate the method. Seven serially diluted LDS concentrations and a buffer (for a negative control) were prepared for a maximum final range from 0 to 150 *μ*M for* h*MAO-A and 0 to 40 *μ*M for* h*MAO-B. Three GST concentrations for a final of 5, 10, and 20 *μ*M and a buffer (for a negative control) were mixed with each isozyme for a final concentration of 0.88 U/mL (2 : 1 ratio). After 40 min, the reactions were initiated by adding 19 *μ*L of the enzyme and GST (or buffer) mixture to LDS in 96-well plates. After 1.5 h of incubation, RLDR was added, and the luminescence signal was detected as mentioned in the* hMAO-A and hMAO-B assay*.

### 2.4. Tyramine Oxidation Assay

A slightly modified continuous spectrophotometric method [27] was used to determine the selectivity of tyramine oxidation by* h*MAO-A or* h*MAO-B inhibited by GST. DEP was used as a standard control for this assay. Briefly, after optimizing tyramine used concentration for* h*MAO-A, 5x substrate and peroxidase chromogen reagent were mixed (1 : 1) for final of 0.5 mM tyramine HCl. In 96-well plates, 25 *μ*L of GST serial dilutions up to 46 *μ*M final concentrations were mixed with 50 *μ*L isozyme for 0.7 U/mL final concentrations (0.09 U per reaction). After 40 min incubation at RT, 50 *μ*L of the reagent/substrate mix was added to initiate the reaction and to make the final concentrations. The developing color (H_2_O_2_ indicator) was monitored over time at 490 nm by the *μ*Quant Microplate Spectrophotometer (Bio-Tek, USA).

### 2.5. Docking Studies

GST, its analog DZ, and a selective MAO-B standard rasagiline (RAS) were docked as test ligands to the X-ray structure of* human* MAO-A and MAO-B at their Ligand Binding Domain. HYBRID of OEDocking (v3.0.1.) by OpenEye Scientific Software (Santa Fe, NM) was used as the docking method [28]. Briefly, MAO-A and MAO-B crystal structures were imported from Protein Data Bank pdf (PDB) to Sybyl-X 1.3 Modeling Suite (Tripos International, St. Louis, MO). Before docking, the conformer ensembles of all the test ligands were generated using OMEGA v2.4.6 (Open Eye Scientific Software, Santa Fe, NM) [28]. Using Structure Preparation tool, MAOs chain A was extracted; each flavin adenine dinucleotide cofactor (FAD) covalently linked to its cysteine residue (CYS: 406: A and CYS: 397: A) was retained in MAO-A and MAO-B, respectively. Hydrogen atoms were added to the isozymes, water molecules were retained, and MMFF94 and MMFF94s force fields were assigned to the atoms. The prediction method was validated by redocking the bound ligands complexes of MAO-A-harmine (PDB: 2Z5X) and MAO-B-2-BFI (PDB: 2XCG) in the crystal structures. The best ten poses retrieved were identical to the original poses of the cognate ligands with root mean square deviation (RMSD) between values <2 Å. Data were presented as HYBRID Chemgauss 4 scores.

### 2.6. Statistical Analysis

All analyses were performed by GraphPad Prism Software 6.02 (San Diego, CA, USA). IC_50_ values were determined by nonlinear regression best-fit model of normalized response with variable slope. The inhibitor constant (*K*_*i*_) values were obtained from the competitive inhibition model. Lineweaver-Burk plot was obtained from Michaelis-Menten data. Relative selectivity (RS) folds were defined by the ratio of* h*MAO-A IC_50_ to* h*MAO-B IC_50_. The significance of the difference between two groups was determined using an unpaired *t*-test, between the control and treatments using one-way ANOVA followed by Dunnett's multiple comparisons tests and between two sets of data using two-way ANOVA followed by Sidak's multiple comparisons test.

## 3. Results

### 3.1. GST MAOs Inhibition Compared with DZ

**Figure 1:**
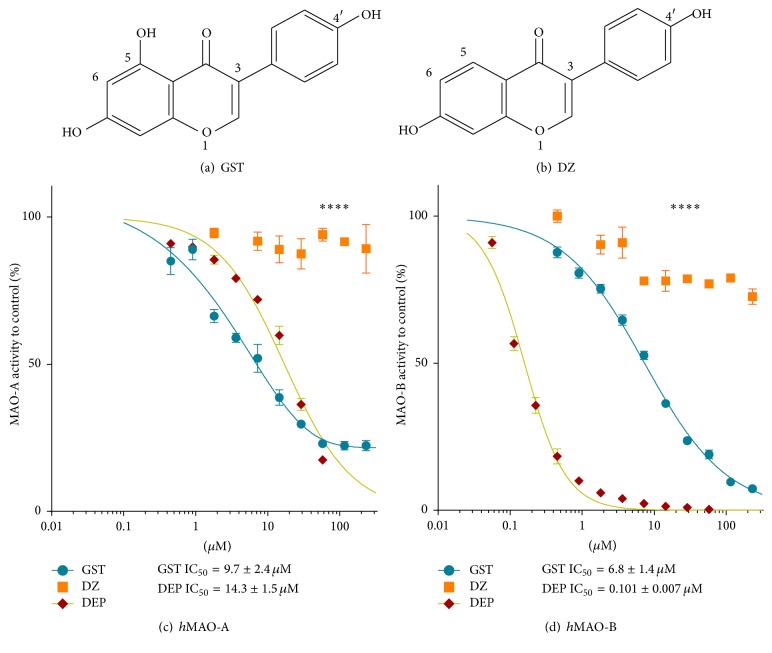
Comparison between (a) genistein (GST) and (b) daidzein (DZ) analogs inhibition of* h*MAO-A (c) and* h*MAO-B (d). Arbitrary Light Units (ALU) were measured at 25°C. Data points were presented as the mean ± SEM, *n* = 3, for two separate experiments. The significance of difference between the isoforms inhibitory effects by GST and DZ was determined using two-way ANOVA followed by Sidak's multiple comparisons test. ^*∗∗∗∗*^ *p* < 0.0001.

The specific inhibitory potentials of GST (Figure 1(a)) on A and B isozymes were investigated and compared to DZ (Figure 1(b)) and DEP using MAO luminescence assay (Figures 1(c) and 1(d)). The data obtained show that GST inhibited* h*MAOs superiorly more than DZ (*p* < 0.0001). DZ showed minor or partial inhibitory effect up to 250 *μ*M of the tested concentrations (*p* < 0.0001). Meanwhile, GST had a sigmoidal inhibitory pattern and potency with an* h*MAO-B mean IC_50_ of 6.81 *μ*M to be 1.4% of DEP potency (Figure 1(d)). However, GST* h*MAO-A inhibitory potency was more potent than DEP by 1.48-fold with mean IC_50_ of 9.7 *μ*M (Figure 1(c)).

### 3.2. Mode of Inhibition of MAO-A and MAO-B Isozymes

#### 3.2.1. Recovery of* h*MAO-A and* h*MAO-B after Inhibition

**Figure 2:**
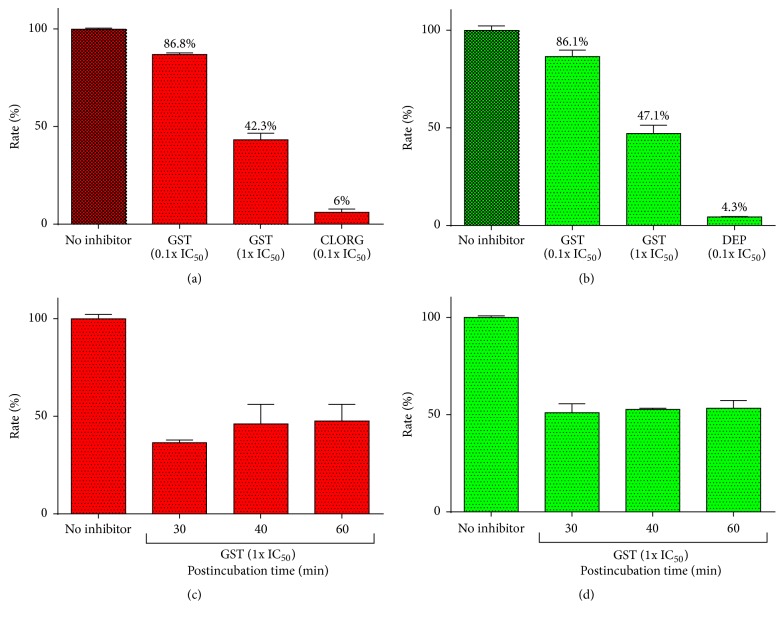
Recovery of the inhibition of recombinant monoamine oxidases,* h*MAO-A (a) and* h*MAO-B (b), by genistein (GST) after 40 min preincubation and GST dilution to 0.1x IC_50_ and 1x IC_50_. The recovery of GST 1x IC_50_ is stable in* h*MAO-A (c) and* h*MAO-B (d). Clorgyline (CLORG) and deprenyl (DEP) were used as standard controls for* h*MAO-A and* h*MAO-B, respectively. The percentage of residual enzymes catalytic rates compared to their related negative control was expressed as mean ± SEM, *n* = 3. Data represent two experiments for each isozyme.

The recovery of the* h*MAO isozymes after GST inhibition was examined to determine reversibility of inhibition. The isozyme activities were determined after enzyme-inhibitor dilution with LDS (Figures 2(a) and 2(b)). At as low as 0.1x IC_50_ value concentrations, irreversible standard inhibitors expectedly reduced the isozyme activities to 6% (CLORG) and 4.3% (DEP), which indicates an unrecovered reduction of MAO activity after incubation with 10x IC_50_ concentrations. Meanwhile, preincubation of 0.1x IC_50_ GST with either* h*MAO-A or* h*MAO-B allowed a recovery of* h*MAO-A and* h*MAO-B catalytic rates to 86.8% and 86.1%, respectively. Their recovered activities measured in the presence of GST at its mean 1x IC_50_ value were 42.3% (MAO-A) and 47.1% (MAO-B). Both isozymes recoveries from GST were close to 90% or 50%, which is expected from a reversible inhibitor. No significant difference was found between the 100x IC_50_ and 4x IC_50_ or between 100x IC_50_ of both preincubated enzyme-inhibitor mixtures (data not shown). To investigate if the inhibition of activity is time-dependent, as it is known for irreversible inhibitors, we measured the postincubation time recovery in Figures 2(c) and 2(d) for* h*MAO-A and* h*MAO-B, respectively. The results obtained provide more evidence that GST is a reversible inhibitor of* h*MAO-A and* h*MAO-B up to the tested 60 min at RT and a maximum of 100x IC_50_ value.

#### 3.2.2. Competitiveness of* h*MAO-A and* h*MAO-B Inhibition

**Figure 3:**
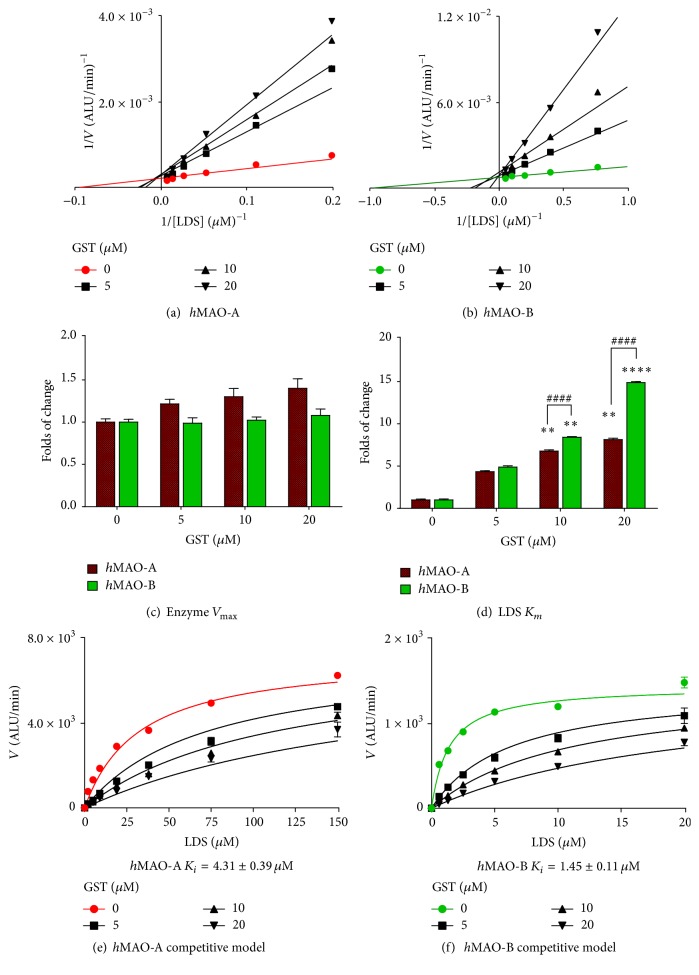
Mode of inhibition of* recombinant human* monoamine oxidases (*h*MAO-A and* h*MAO-B) by genistein (GST) compared to control the initial velocity (*V*). Lineweaver-Burk plots for* h*MAO-A (a) and* h*MAO-B (b) with a gradual increase of luciferin derivative substrate (LDS). Linear regression data are presented as mean ± SEM of *n* = 3. The maximum velocity (*V*_max_ ± SEM) (c) and Michaelis constant (*K*_*m*_ ± SEM) of LDS (d) parameters folds of change were measured with a gradual increase of GST concentrations in both isozymes. GST inhibitor constant (*K*_*i*_) was determined using the competitive inhibition model for* h*MAO-A (e) and* h*MAO-B (f) as the best fit. Regression data are presented as the mean ± SEM of *n* = 3. The significance of difference between the controls and treatments was determined using one-way ANOVA followed by Dunnett's multiple comparisons test and between two groups of each concentration using *t*-test. ^*∗∗*^ *p* < 0.01;  ^*∗∗∗∗*^ or ^####^ *p* < 0.0001.

Further investigations on the competitiveness of GST reversible inhibition on both MAOs are illustrated in Figure 3. The Michaelis-Menten kinetics for both isozymes at their predetermined initial rates of reaction are presented as Lineweaver-Burk plot (Figures 3(a) and 3(b)). The intersection of all linear regression lines at *y*-axis and not *x*-axis indicates a competitive mode of inhibition. Meanwhile, DEP linear regression crossed *y*-axis with hMAO-A and *x*-axis with MAO-B, confirming its noncompetitive* h*MAO-B mode of inhibition (data not shown). *V*_max_ and LDS *K*_*m*_ in the presence of GST were further determined and illustrated as folds of change (Figures 3(c) and 3(d)). GST showed no significant change in *V*_max_ values (*p* = 0.10 for* h*MAO-A and *p* = 0.44 for* h*MAO-B) while LDS *K*_*m*_ values increased severalfold with GST increased concentrations (*p* < 0.002 for* h*MAO-A and *p* < 0.0001 for* h*MAO-B). Interestingly, GST increased LDS *K*_*m*_ for* h*MAO-B more significantly (14.9-fold) than LDS *K*_*m*_ for* h*MAO-A (8.1-fold) by more than 1.8-fold at 20 *μ*M GST concentration.

To further assess the GST competitiveness, the alpha parameter was determined. The alpha value is used in Prism software to determine the degree at which the inhibitor can change the affinity with increasing substrate concentration. In the mixed model of inhibition analysis, alpha values were “too large” in all GST concentrations and both isozymes. The large alpha value indicates that GST reduced the substrate-binding affinity to the isozyme. The above studies exclude the noncompetitive, uncompetitive, or even mixed mode of GST inhibition to favor a competitive mode of inhibition on both MAOs.

The competitive behavior model was used for the determination of GST inhibition constant (*K*_*i*_) for both isozymes by Prism (Figures 3(e) and 3(f)). GST in the competitive model of inhibition of* h*MAO-A and* h*MAO-B activities had the best-fit *R* ^2^ of 99% and 97%, respectively. GST mean* h*MAO-B *K*_*i*_ of 1.45 *μ*M (Figure 3(f)) was lower than* h*MAO-A *K*_*i*_ of 4.31 *μ*M (Figure 3(e)) by 3.0-fold. The lower *K*_*i*_ indicates lower GST concentration needed to reduce* h*MAO-B activity rate to 50%.

#### 3.2.3. Tyramine Selective Oxidation by MAOs

**Figure 4:**
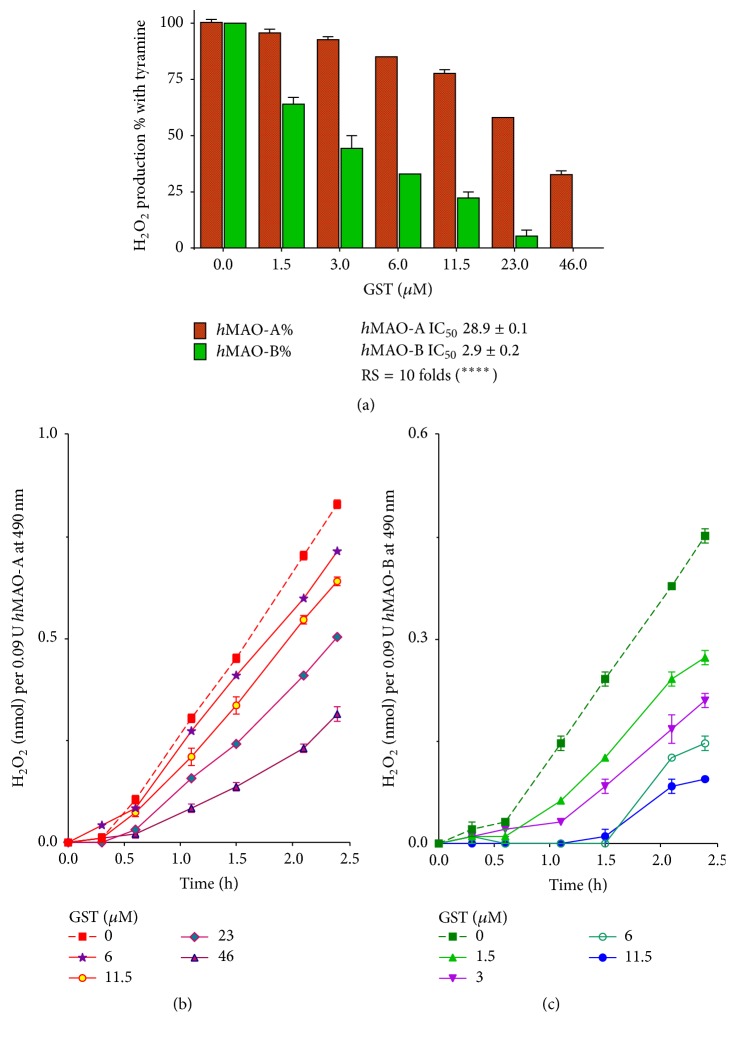
Genistein (GST) inhibited* human* monoamine oxidases (*h*MAO-A and* h*MAO-B isozymes) H_2_O_2_ byproduct formation differently (^*∗∗∗∗*^ *p* < 0.0001) by using tyramine as a substrate (a). The enzymatic reaction was monitored in* h*MAO-A (b) and* h*MAO-B (c) without GST (dotted line) or in the presence of different GST concentrations at RT. IC_50_s and data points were presented as the mean ± SEM, *n* = 3. RS is relative selectivity to inhibit B isozyme. The significance of difference of RS against hMAO-B was determined using unpaired *t*-test.

Tyramine oxidation MAO assay was conducted to define RS of GST for* h*MAO-A and* h*MAO-B inhibition (Figure 4). Separate continuous* h*MAO-A and* h*MAO-B tests have shown that H_2_O_2_ was produced time-dependently at RT and was inhibited by DEP and CLORG standards (data not shown). After 2 h incubation, GST inhibited the H_2_O_2_ production by* h*MAO-B more selectively (IC_50_  2.9 ± 0.2 *μ*M) than of* h*MAO-A (IC_50_  28.9 ± 0.1 *μ*M) by about 10-fold (*p* < 0.0001) when using tyramine as a substrate (Figure 4(a)). GST inhibited* h*MAO-B H_2_O_2_ byproduct formation more potently and time-dependently than* h*MAO-A H_2_O_2_ (Figures 4(b) and 4(c)).

### 3.3. Docking Studies

**Figure 5:**
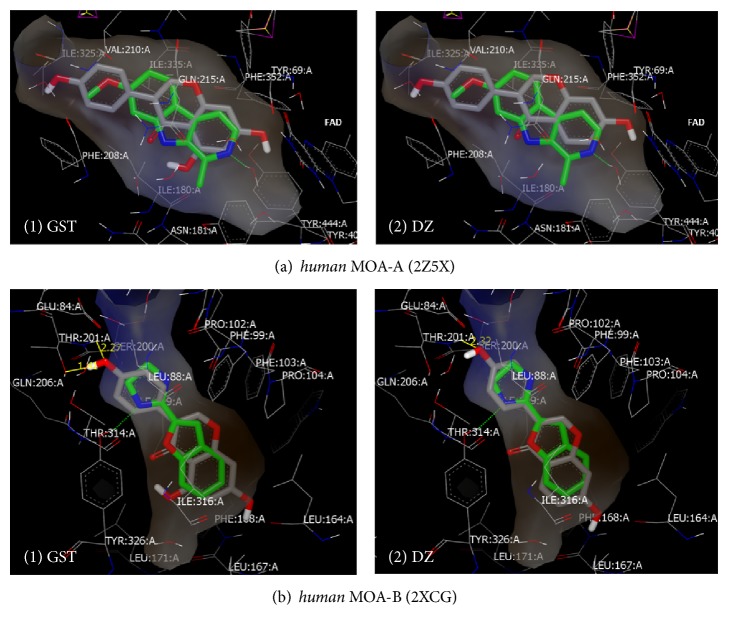
Predicted docking orientations of genistein (GST) and its analog daidzein (DZ) at* human* monoamine oxidase-A (MAO-A) (a) and MAO-B (b) active sites. ((a)(1)) GST and ((a)(2)) DZ with harmine (2Z5X) within MAO-A. ((b)(1)) GST and ((b)(2)) DZ with 2-(2-benzofuranyl)-2-imidazoline (2XCG) within MAO-B. Key: the hydrophobic zones are in brown color, the neutral zones are in gray, the hydrophilic zones are in blue, docked standard ligands are in green, and tested compounds are in gray.

Both analogs of GST and DZ were successfully docked to the same* human* MAO-A and MAO-B crystal structure active sites at which standards of RAS, 2Z5X, and 2XCG interacted. Affinity scores and orientation predictions results are illustrated in Figure 5 and Table 1. In Figure 5, GST and DZ in MAO-A and MAO-B shared close docking poses orientations. In the* human* MAO-A model, GST and DZ within the MAO-A active site cavity in Figure 5(a)(1) and 5(a)(2) were illustrated with 2Z5X. Their chromone moieties were located in the compact entrance cavity and close to the FAD, but their hydroxy-phenyl moiety was attracted to the hydrophobic active site entrance surfaces (brown zones). Orientations of both isoflavones matched and crossed with 2Z5X standard orientation. However, a slight pull of GST toward a hydrophilic zone at its C5-OH group gave a better-matched pose to the standard without H-bonds predictions.

In the* human* MAO-B model with 2XCG (Figure 5(a)(1) and 5(a)(2)), the substrate cavity structure is illustrated with both GST and DZ isoflavones. Similarly, both analogs chromone moieties were located entirely in the hydrophobic zone of the active site cavity in the substrate-binding domain (brown zone). Both analogs were away from FAD or its surrounding tyrosine residues by having their hydroxy-phenyl moiety close to the entrance cavity. However, GST C4′-OH group moiety had more H-bonds predictions away from the hydrophobic residues than DZ. That orientation may enhance more reversibility by not affecting the FAD structure and having reversible hydrophobic and H-bond interactions.

On the other hand, the affinity of the active GST and the partially active DZ were predicted as docking scores (Table 1). The analogs were compared to RAS instead of DEP because it showed H-bond interactions while DEP did not (data not shown). The affinities were similar to both isozymes including the type of H-bond interactions formed with MAO-B. The analogs' high MAO-B affinity scores (>1.8-fold MAO-A scores) were concomitants of the prediction of H-bond interactions between threonine (THR: 201: A) and analogs' oxygen donor and acceptor of C4′-OH which acted as an acceptor here. Although their interaction distances were similar (2.27 and 2.32 Å, resp.), the same oxygen in GST served as a donor and a stronger extra H-bond was predicted with threonine (1.41 Å), making the distances comparable to 2XCG. However, predicted affinities, site, and type of H-bond interactions were different with the standard ligands.

## 4. Discussion

The two natural isoflavone analogs GST and DZ showed a dramatic difference in their MAO inhibitory properties with GST being highly more potent than DZ. The chemical structure similarities of DZ to GST enabled us to explain GST MAO-A and MAO-B inhibitory actions. With using the chemical analog DZ, the structure-activity relationship between their functional groups and* h*MAO-A and* h*MAO-B inhibitions indicates that it is not the lipophilic chromone-core structure of GST and DZ causing GST MAOs inhibitory activities. Rather, the C5-OH extra group ruled the observed activity. The obtained data (Figure 1) highlights the role of C5 substitution in the isoflavones chromone-core structure on* h*MAOs inhibition. The data further suggest a crucial role of C5-OH and the threonine 201 of MAO-B in MAOs inhibitory action and selective inhibition. GST was also compared with DEP, the standard therapy for PD recently used in MDD [6, 16]. The obtained results indicated GST higher potency than DEP to inhibit* h*MAO-A with much less* h*MAO-B selectivity. However, GST had a reversible and competitive MAO inhibition with higher affinity to MAO-B than MAO-A. That encouraged us to propose the use of GST for PD with depression.

In contrast to the irreversible inhibitors DEP and CLORG, GST allowed the recovery of both* h*MAO-A and* h*MAO-B isozymes time-independently (Figure 2). Furthermore, the Lineweaver-Burk linear regression and the severalfold increase of LDS *K*_*m*_ clearly indicated GST competitive mode of inhibition for both* h*MAO isoforms (Figure 3). A similar MAO mode of inhibition by other flavonoids was previously reported [29, 30]. The GST competitiveness with the substrate confirms its reversible mode of inhibition [31]. Therefore, GST* in vivo* withdrawal may easily recover both isozymes activities. Likewise, recovery of isozymes may occur with an occasional sharp increase of tyramine, DA, NE, and 5-HT, which is crucial in reducing undesirable associated adverse effects [32].

Although LDS binding affinity to* h*MAO-B is higher than* h*MAO-A [25], with GST, LDS *K*_*m*_ was increasing more sensitively in* h*MAO-B than* h*MAO-A (Figure 3(d)). The obtained results of* h*MAO-A GST *K*_*i*_ values are found to be close to our previously reported DEP *K*_*i*_ of 3.1 *μ*M reported using similar conditions [30]. However, the GST *K*_*i*_ value in* h*MAO-A is still higher than* h*MAO-B. Those results indicate GST higher competitiveness and binding affinity to* h*MAO-B more than* h*MAO-A. In the presence of tyramine substrate (Figure 4), GST needed 10-fold of* h*MAO-B inhibitory concentrations to inhibit* h*MAO-A activity equivalently. GST* h*MAO-B inhibition selectivity is consistent with Figure 3 results and may have increased by tyramine's better affinity to* h*MAO-A than* h*MAO-B [25]. Thus, GST may allow tyramine metabolism by uninhibited* h*MAO-A without losing the capability to diminish MAO-B H_2_O_2_ and aldehydes cytotoxic byproducts.

To understand the molecular interactions involved in GST affinity and the C5-OH role of inhibition, we conducted a molecular docking study for both analogs. Both analogs matched the standards orientations at the isozymes entrance cavities with identical high affinity-binding predictions (Figure 5 and Table 1). The attractiveness of C5-OH to MAO-A hydrophilic zone may have contributed to GST reversible inhibition. However, the analogs' isoflavone structure may form more stable lipophilic interactions with MAO-B highly lipophilic zones than MAO-A. In addition, unlike MAO-A, MAO-B constitutes high H-bonding forming polar residues that surround the entrance cavity outer part [33]. The MAO-B acceptor and donor H-bonds may improve flavonoids affinity scores or/and inhibition [34] which may also depend on the site and strength of interaction. The formation of two acceptor and donor H-bonds between threonine and GST may have led to the complete MAO-B inhibition. Consistently in our previous report, flavonoid bavachinin was predicted to bind to threonine 201 and showed close* h*MAO-B inhibitory activity [30]. That may underline the importance of threonine contribution in flavonoid inhibitory activity. The obtained results emphasize C5-OH role in stronger H-bonds formation with threonine but eliminate its effects on increasing affinity. Thus, with the assumption that the C5-OH (the absent group in DZ) is required for GST inhibitory role, GST can bind to MAO-B better than MAO-A with the strength to impede LDS and tyramine from binding and theoretically reduce its H_2_O_2_ byproduct in the brain.

On the other hand, overly active and/or highly expressed MAOs contribute significantly to oxidative stress, mitochondrial toxicity, DNA damage, lipid peroxidation, and cancer [35, 36] in addition to the catalysis of neurotoxic production from MPTP [37]. Meanwhile, GST has been reported to have anti-inflammatory, antioxidant, anticancer effects [38–40] and induced the different antioxidant enzymes [41].* In vitro*, GST had been protected against H_2_O_2_-induced cellular DNA damage in an aging-related prostate cancer [42] and reduced Amyloid *β*- and H_2_O_2_-induced neurotoxicity in human neuroblastoma cells [43]. Moreover, GST protected the dopaminergic neurons and enhanced Bcl-2 gene expression in the MPTP-induced PD mice model [44], which may indicate its ability to cross the blood-brain barrier and inhibit MAO-B, since its bioavailability is reasonable in human [45]. Clinically, GST oral administration improved cognitive functions and memory in males and females [46, 47].

The determination of the proper dosage in patients to achieve MAOs inhibitory concentrations of GST based on the current* in vitro* study needs more research at the basic and the clinical levels. Considerations of GST metabolism and pharmacokinetics should be taken. In a clinical investigation using oral GST to manage postmenopausal osteoporosis in women, a dose of 54 mg/day was needed to achieve a steady-state level up to 1.00 *μ*M [48]. We believe that the required dose of GST to achieve adequate therapeutic MOA inhibition might be less than the extrapolated dose available in the literature for three reasons. Firstly, GST was reported to be metabolized to its precursor biochanin A by intracellular enzymes [49, 50]. In our laboratory, we have found that biochanin A is more potent and selective* h*MAO-BI with an IC_50_ of 0.09 *μ*M and an* h*MAO-A IC_50_ of 3.4 *μ*M (Zarmouh et al., data to be published). Secondly, the dose of GST can be more than 54 mg/day as reported in a clinical trial in elderly [51]. Thus, both GST and metabolites may reach their target in the brain. Thirdly, we compared the MAOI of GST to DEP that needs higher doses for MAO inhibition. DEP was first investigated as an antidepressant but was not used because of its low bioavailability and the need for high oral doses as an MAO-AI [52] (greater than GST). DEP low bioavailability is mainly due to its high first-pass metabolism to amphetamine metabolites [6]. Nonetheless, DEP was formulated for skin delivery as DEP (Emsam) and, consequently, became a well-established effective MAOI used for depression in the elderly as a transdermal patch. This route of administration allowed higher bioavailability and also fewer restrictions on diet [53]. Similarly, GST bioavailability could be improved using the transdermal patch. Therefore, clinical and pharmacokinetic parameters investigations are needed to search for the best GST preparations or route of administration to achieve effective therapeutic concentrations in the brain for MAOs inhibition.

## 5. Conclusions

The obtained results indicate that the isoflavone GST has potent* h*MAO-A and* h*MAO-B inhibitory activities compared to its analog DZ negligible inhibitory effects. GST MAO-A inhibition potency was more than the standard DEP. However, its affinity and potency to inhibit* h*MAO-B were higher than* h*MAO-A. GST valuable MAO inhibitory capacity over DZ and selectivity to inhibit MAO-B may be due to the presence of C5-OH substitution that may have worked as an inhibitory group. The data obtained clearly show that GST is a reversible and competitive MAO-A and MAO-B inhibitor and, consequently, can safely decrease* h*MAOs toxic H_2_O_2_ byproduct, with the least chance to have the cheese effect incidence. Based on the data obtained in this study we recommend further investigations to examine the use of GST as a possible agent for the therapeutic management of PD patients with depression.

## Figures and Tables

**Table 1:** Docking scores of GST and DZ within X-ray *human* monoamine oxidase-A and -B (MAO-A and MAO-B) active sites, in comparison with MAO selective inhibitors.

Ligand^a^	MAO-A		MAO-B		Å	Type^c^	Amino acid
	Docking score^b^	H-bonds predicted	Docking score^b^	H-bonds predicted			
DZ	−6.9	0	−12.8	1	2.32	O⋯HN	THR: 201: A
GST	−7.0	0	−12.8	2	2.27	OH⋯N	THR: 201: A
					1.41	O⋯HN	THR: 201: A

RAS	−8.8	0	−10.7	1	2.22	NH⋯O	PRO: 102: A
2Z5X	−8.4	0^*∗*^	−11.6	1	1.76	NH⋯O	PRO: 102: A
2XCG	−12.7	0	−9.8	2	1.32	NH⋯O	PRO: 102: A
					2.24	N⋯HO	TYR: 326: A

^a^Ligands docked: daidzein (DZ); genistein (GST), with reported standards; rasagiline (RAS); harmine (2Z5X); 2-(2-benzofuranyl)-2-imidazoline (2XCG).

^b^HYBRID Chemgauss 4 scores.

^c^The type of H-bond between the ligand and the amino acid active site.

^*∗*^H-bond formed between the ligand and the water molecule active site.

## Abbreviations

2-BFI: 2-(2-Benzofuranyl)-2-imidazoline
5-HT: Serotonin
2XCG: 2-(2-Benzofuranyl)-2-imidazoline-MAO-B complex
2Z5X: Harmine-MAO-A complex
AD: Alzheimer's disease
ALU: Arbitrary Light Units
CLORG: Clorgyline
DA: Dopamine
DEP: (R)-(−)-Deprenyl HCl
DZ: Daidzein
FAD: Flavin adenine dinucleotide
GST: Genistein
H_2_O_2_: Hydrogen peroxide
HBSS: Hank's Balanced Salt Solution
*h*MAO: * Recombinant human* MAO
*h*MAO-AIs: * Recombinant human* MAO-A inhibitors
*h*MAO-BIs: * Recombinant human* MAO-B inhibitors
*K*_*i*_: Inhibitor constant
*K*_*m*_: Michaelis constant
LDS: Luciferin derivative substrate
MDD: Major depressive disorder
MAO: Monoamine oxidase
MAOIs: MAO inhibitors
MPTP: 1-Methyl-4-phenyl-1,2,3,6-tetrahydropyridine
NE: Norepinephrine
PD: Parkinson's disease
PDB: Protein Data Bank pdf
RAS: Rasagiline
RLDR: Reporter luciferin detection reagent
RS: Relative selectivity
*V*_max_: Maximum velocity.
